# Effectiveness of Transcranial Magnetic Stimulation on Executive Function, Attention, and Memory in Stroke Patients: A Systematic Review and Meta-Analysis

**DOI:** 10.7759/cureus.75194

**Published:** 2024-12-06

**Authors:** Ha T Le, Kenta Honma, Hiroki Annaka, Sun Shunxiang, Tsukasa Murakami, Tamon Hiraoka, Tomonori Nomura

**Affiliations:** 1 Department of Rehabilitation, Hai Duong Medical Technical University, Hai Duong, VNM; 2 Graduate School, Niigata University of Health and Welfare, Niigata, JPN; 3 Department of Occupational Therapy, Faculty of Rehabilitation, Niigata University of Health and Welfare, Niigata, JPN

**Keywords:** attention, cognitive impairment, executive function, memory, meta-analysis, stroke, systematic review, transcranial magnetic stimulation

## Abstract

Transcranial magnetic stimulation (TMS) is an effective intervention for improving cognitive impairment in patients with stroke. However, its effectiveness in the subdomains of cognition is conflicting and not clearly established. This systematic review assessed the efficacy of TMS in improving executive function, attention, and memory in this population. Seven databases, including PubMed, Scopus, Cochrane Library, Cumulated Index in Nursing and Allied Health Literature, NeuroBITE, Physiotherapy Evidence Database, and OTseeker, were searched for indexed literature until July 2024 to identify all randomized controlled trials (RCTs) of this effect in stroke patients. This systematic review was performed by Preferred Reporting Items for Systematic Reviews and Meta-Analyses guidelines and the Handbook of the Cochrane Library and evaluated the quality of evidence using the Risk of Bias 2 tools and grading of recommendations assessment, development, and evaluation (GRADE) systems. Meta-analyses were performed using standardized mean difference (SMD) (Hedge's g) as the effect measure, and subgroups were performed to explore potential outcomes. The research included 13 RCTs involving 496 patients with stroke. The results indicated that TMS could affect executive function (six RCTs with SMD = 0.55; 95% confidence interval, CI = 0.04-1.05) and memory (nine RCTs with SMD = 0.57; 95% CI = 0.25-0.89) in patients with stroke. However, the effectiveness of TMS on attention (five RCTs with SMD = 0.32; 95% CI = -0.1 to 0.75) was not clear. The quality of the results varied between very low and low according to the GRADE approach. In conclusion, TMS may affect executive function and memory, but not attention. The quality of the evidence for the outcomes varied from very low to low owing to heterogeneity and bias; therefore, the results should be considered with caution, and more rigorous evidence is needed.

## Introduction and background

Cognitive impairment in stroke patients is a common problem that has profound effects on the function, dependency, quality of life, and mortality of patients, as well as the burden on family and society [1-6]. The reported rate of cognitive impairment after a stroke is 20%-80% [2,7,8]. Cognitive impairment has many components, including problems with executive function, attention, memory, orientation, language, and global cognitive functioning [8]. With advancements and expertise in medicine, cognitive issues are investigated and evaluated in depth and detail, specifically to identify the subdomains and components of defects [9-11]. Therefore, a more intensive, specific, and effective intervention plan is needed for stroke patients with cognitive impairment. Traditional rehabilitation is commonly used to treat cognitive impairment [12,13]. Currently, there are some notable methods with many advantages, such as rehabilitation robots, repetitive transcranial magnetic stimulation, brain-computer interfaces, augmented reality, transcranial direct current stimulation (tDCS), and virtual reality [14-17]. Transcranial magnetic stimulation (TMS) is an effective intervention [18].

TMS is a noninvasive neurostimulation [19] that helps 1) stimulate and inhibit neuronal connections and induce changes in conduction and synaptic plasticity, 2) stimulate specific genetic expression, and 3) change the morphology of neurons and induce brain neurogenesis [20-22]. TMS included high-frequency TMS (HF-TMS), low-frequency TMS (LF-TMS), and theta burst stimulation (TBS) [20]. The LF-TMS protocol, composed of pulses of ≥1 Hz, may cause inhibition effects, but the HF-TMS protocol involves pulses of ≥5 Hz, resulting in a stimulation effect to change the excitability of the cortex [23]. TBS is a three-phase burst with a frequency of 50 Hz every 200 ms and is usually used in two main patterns: continuous TBS and intermittent TBS (iTBS) [24]. Depending on the type, size, shape, direction of the coils, frequency, and strength of the magnetic pulses, selective inhibition or stimulation of neurons may produce neuronal plasticity [25]. In stroke patients, TMS is a potential method not only for cognitive impairment but also for other problems, including motor function, activities of daily living, poststroke depression, dysphagia, spasticity, and quality of life [18,26-37]. Moreover, TMS can cause long-term brain effects [35,38] and is well-tolerated, safe, and effective in stroke therapy [18,25,28,35,39].

In cognitive impairment, executive dysfunction, attention deficits, and memory deficits are among the most common and prevalent problems [9,40,41], greatly affecting the patient's functioning, reintegration, and quality of life [40,42]. TMS is effective in treating cognitive impairment in stroke patients in general [33,39,43]; however, for subdomains, specifically executive function, attention, and memory, to the best of our knowledge, there is currently no consensus or clear evidence of the effectiveness of TMS's effectiveness in these domains [12,13,32,44,45].

This study aimed to discover the effectiveness of TMS on each subdomain of cognitive impairment, including executive function, attention, and memory, to provide suggestions for specific clinical treatment and more effective application of TMS for stroke patients with damage in different subdomains of cognitive impairment.

## Review

Methods

This systematic review was conducted in accordance with the Cochrane Handbook for Systematic Reviews of Interventions [46] and the Preferred Reporting Items for Systematic Reviews and Meta-Analyses (PRISMA) guidelines [47,48]. The review protocol was registered in the International Prospective Register of Systematic Reviews under the registration number CRD42024549230 [49].

Search Strategy

We systematically searched seven databases, including PubMed, Scopus, Cochrane Library, Cumulated Index in Nursing and Allied Health Literature, Physiotherapy Evidence Database, OTseeker, NeuroBITE, and gray literature. The searches were performed on articles indexed before April 24, 2024, and then updated until July 31 to identify English-related articles on our subject. We also searched for references to the full-text articles that were included.

Search strategies were based on the problem, intervention, comparison, and outcome tool [46]: 1) problem: stroke with cognitive impairment (executive function, attention, and memory); 2) intervention: TMS; 3) comparison: control group including sham, cognitive training, tDCS, or other therapies; and 4) outcome: any possible result related to executive function, attention, and memory. Each database was searched for control vocabulary and free-text terms. The entire search strategy is presented in Appendix 1.

Study Selection and Inclusion Criteria

Two authors (H.T.L. and K.H.) independently conducted the study selection process and evaluated the inclusion criteria by screening titles, abstracts, and full texts. Disagreements during the selection and screening process were resolved by a third author (T.N.).

The inclusion criteria were 1) randomized controlled trials (RCTs); 2) RCTs with a sample of patients diagnosed with stroke, who had cognitive impairment or deficits in executive function, memory, or attention, and had outcome measures related to these outcomes; 3) studies that used TMS (HF, LF, and theta-bust) as an intervention (alone or combined with other therapy); and 4) English-language RCTs. The exclusion criteria were RCTs with participants who had a stroke and other diseases and studies without a full text.

Data Extraction

Two authors independently extracted the data from each included study. A third reviewer was consulted to resolve any disagreements. Furthermore, we attempted to emulate the authors of the included studies for more information and input data as needed. Data obtained from each study were collected by two authors using a standardized form of Microsoft Excel (Microsoft Corporation, Redmond, WA). Each study collected the following data: 1) general characteristics, including the year of publication, authors, study design, country, sex, age, stroke type, onset, sample size, level of disability (cognition and motor), injury brain site, inclusion criteria, and the number of groups; 2) intervention characteristics in experimental and comparison groups, encompassing the type of intervention (high, low, iTBS), coil type, intensity, stimulation position, adverse effect, and dose (pulses, minutes of each session, days, weeks, total of sessions); and 3) results were collected, encompassing the outcome measurements, related quantitative data, and time follow-up. Outcome measurements were collected after the included studies and evaluation of the results.

For importing data, deleting duplicates, and screening titles and abstracts, the Rayyan [50] website was used, Microsoft Excel was used for data collection, Microsoft Word was used to create table syntheses, and Endnote was used to manage the studies and references included.

Statistical Analysis

The meta-analyses were conducted by two authors (T.M. and H.T.L.) using R, version 4.3.1 software (meta and metafor package) with the guidance of from work by Harrer et al. [51]. To assess the outcomes, the standardized mean difference (SMD), Hedge's g, and its 95% confidence interval (95% CI) were used as effect measures. In each study, the web Campbell Collaboration was used to calculate SMD [52] based on the mean and SD pre- and postintervention, or in a study, data were estimated from the figure (Loewenstein Occupational Therapy Cognitive Assessment, LOTCA-attention) [53]. In some RCTs, if many scales were used to assess one outcome in a study, we used the pooled effect size (ES) to obtain a representative ES using the metafor package (R software, R Foundation for Statistical Computing, Vienna, Austria) for meta-analysis. Studies with nonnormal distributions were excluded from the meta-analysis. Hedge's g was used to calculate the pooled SMD [54], and the SMD values were indicated as large (SMD = 0.8), moderate (SMD = 0.5), and small (SMD = 0.2) [55] and were displayed in a forest plot. We used a random-effects model for the meta-analysis; with low heterogeneity (<50%) or consensus in SMD values, a fixed-effect model was used. Heterogeneity was evaluated using the I² statistic, and an I² > 50% was considered heterogeneous throughout the study. Subgroups (according to time phase and type of TMS) were analyzed to explore the potential results.

Assessment of the Risk of Bias and the Quality of Evidence

Two reviewers independently assessed the methodological quality of our findings and the risk of bias. By using version 2 of the Cochrane Risk of Bias Tool for Randomized Trials, the risk of bias in each study was evaluated with the classifications as "high risk," "some concerns," and "low risk," assessing five domains (randomization process, deviations from the intended interventions, missing outcome data, measurement of the outcome, and selection of the reported results) [56]. In terms of the overall quality of evidence for each outcome, the Grading of Recommendations Assessment, Development, and Evaluation (GRADE) system was used to analyze various aspects [57,58]. Any disagreement regarding the judgment of the level of evidence was resolved by a third author.

Publication Bias

This systematic review was conducted because of the variations in the results of the 13 included RCTs. For each outcome, a meta-analysis was performed with five, six, or nine studies, and funnel plots were not created because the number of RCTs was insufficient [46].

Results

Study Selection

**Figure 1:**
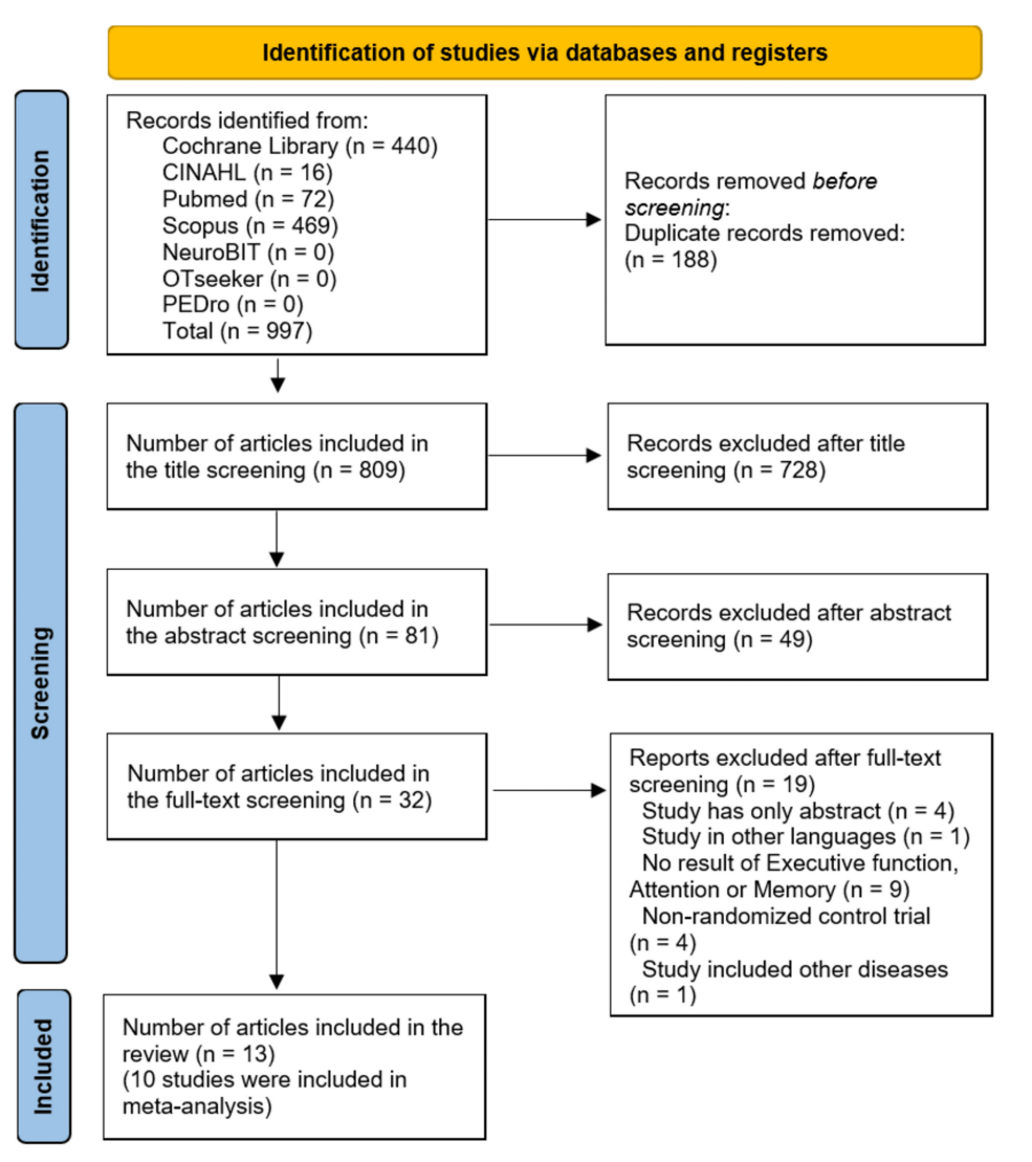
PRISMA flowchart for the systematic review PRISMA: Preferred Reporting Items for Systematic Reviews and Meta-Analyses Image credits: This is an original image created by the author Ha T. Le

Search results and screening stages are shown in the PRISMA guideline flowchart (Figure 1). First, 997 studies were identified from seven databases. After deleting duplicates and checking the titles and abstracts, 32 full texts were included. After screening full texts and emailing authors (when more data were needed), 19 studies [59-77] were excluded, and the remaining 13 trials that met the inclusion criteria were included in the review.

Characteristics of the Studies Included

The 13 trials (Table 1) included 496 participants aged 18-89 years. Of these, nine RCTs were performed in China. Seven studies reported that 55.6% and 88.2% of participants were men. In the included trials, almost all included stroke patients were from the subacute and chronic phases; for time of onset, four RCTs included patients under three months, two RCTs with less than six months, four RCTs with less than one year, and three RCTs with population had a time of onset of more than one year. There were 10 RCTs comparing the TMS group with the sham group, three RCTs with the control group (cognitive training), and one RCT compared with tDCS. In these studies, eight RCTs were conducted with two groups and five RCTs with three groups. Almost all the RCTs included stroke, whether left or right hemispheric, ischemic, or hemorrhagic cases. Eleven of the 13 RCTs included patients with cognitive impairment at the beginning, and two RCTs did not consider cognitive impairment as one of the inclusion criteria.

**Table 1:** Characteristics of the 13 studies included Cog: cognitive; cog-reha: cognitive rehabilitation; ds: days; F: frequency; HF: high frequency; HF-TMS: high-frequency transcranial magnetic stimulation; iTBS: intermittent theta-burst stimulation; LDLPFC: left dorsolateral prefrontal cortex; LF: low frequency; LF-TMS: low-frequency transcranial magnetic stimulation; MMSE: Mini-Mental State Examination; MoCA: Montreal Cognitive Assessment; MT: motor threshold; NI: no information; R/L: right/left; RBANS: Assessment of Neuropsychological Status; RBMT: Rivermead Behavior Memory Test; RDLPFC: right dorsolateral prefrontal cortex; rTMS: repetitive transcranial magnetic stimulation; tDCS: transcranial direct current stimulation; TMS: transcranial magnetic stimulation; WAIS: Wechsler Adult Intelligence Scale; ws: weeds

Characteristics								Intervention					
Study	Country	Time of onset	Injury site (brain) R/L	Types of stroke (I/H)	Severity (physical/cognitive)	Age and sex (%male)	Groups (n)	Pulse, weeks, sessions	Coil	%MT	Frequency (Hz)	Stimulation site	Adverse effect
Najafabadi et al. [78]	Iran	>1 year	Left	Stroke	The Digit Span (subWAIS-memory) minimum score (8 ± 2)	Age: 55-75; sex: 55.6%	1) TMS + computer-based cognitive therapy (n = 9); 2) computer-based cognitive therapy (n = 9)	600, 5ws, 15 ses	NI	100%	HF-TMS (10 Hz)	LDLPFC (F3)	NI
Hu et al. [79]	China	<6 months	NI	Stroke	MoCA < 26 and RBMT ≤ 21	Age: Sham 61.5 ± 9.1; rTMS 63.9 ± 6.3; rTMS-tDCS 64.5 ± 7.2; sex: 88.2%	1) TMS (n = 12); 2) TMS-tDCS (n = 10); 3) Sham (n = 12)	1,200, 4ws, 20	O	80%	HF-TMS (5 Hz)	LDLPFC (F3)	No significant adverse effects
Kim et al. [80]	Korea	>1 year	R/L/multiple 12/4/2	14/4	Korean MMSE: 10-24	Age: 55-70; sex: 55.6%	1) LF-TMS (n = 6); 2) HF-TMS (n = 6); 3) sham (n = 6)	450-HF 900-LF, 2ws, 10	8	80%	LF-TMS (1 Hz) HF-TMS (10 Hz)	LDLPFC (F3)	No major side effects
Zhang et al. [81]	China	<1 year	NI	Stroke	MMSE < 26	Age iTBS: 58 ± 14.6; control: 66.9 ± 12.9; sex: 76.7%	1) iTBS + cognitive training (n = 19); 2) control: cognitive training (n = 18)	600, 6ws, 30	8	70%	iTBS	LDLPFC (F3)	NI
Yu et al. [82]	China	<3 months	7/11	10/8	MoCA 15-25; walk > 10 meters independently	Age: rTMS 54.6 ± 11.8; Sham 57.4 ± 12.8; sex: 83.3%	1) TMS + routine (n = 9); 2) rehabilitation treatment (n = 9); 3) Sham + routine rehabilitation treatment	1,200, 2 ws, 10	8	80%	HF-TMS (5 Hz)	LDLPFC	NI
Yin et al. [83]	China	<6 months	10/13/11 (left/right/bilateral)	23/11	MoCA < 26	Age: 30-75; sex: 88.2%	1) TMS + Cog-reha (computer) (n = 16); 2) Sham + Cog-reha (computer) (n = 18)	2,000, 4ws, 20	8	80%	HF-TMS (10 Hz)	LDLPFC	NI
Liu et al. [84]	China	<1 year	33/25	35/23	Attention dysfunction (MMSE). Good motor function	Age: 40-75; sex: 44.8%	1) TMS + Cog training (n = 29); 2) Sham + Cog training (n = 29)	700, 4ws, 20	8	90%	HF-TMS (10 Hz)	LDLPFC (F3)	NI
Chu et al. [53]	China	<1 year	23/37	39/21	MMSE: mild to severe	Age: iTBS 57.2 ± 14.3; tDCS 61.6 ± 14.2; control: 66.8 ± 12.2; sex: 75%	1) iTBS + Cog Training (n = 21); 2) tDCS + Cog Training (n = 19); 3) Control Group (Cog Training) (n = 20)	600, 6ws, 30	8	70%	iTBS	LDLPFC	NI
Tsai et al. [85]	Taiwan	<3 months	Left	20/21	RBANS: 24-85	Age: 5 Hz: 57.5 ± 12.3; iTBS: 60.1 ± 14.1; sex: 80.5%	1) HF-TMS (n = 11); 2) iTBS (n = 15); 3) sham (n = 15)	600 (iTBS, HF), 2ws, 10	8	80%	HF-TMS (5 Hz) iTBS	LDLPFC	No seizure or other adverse effects
Li et al. [86]	China	<3 months	18/40	32/26	Cog impairment by MMSE	Age: 18-65; sex: 58.6%	1) iTBS + Cog-reha (n = 28); 2) sham + Cog-reha (n = 30)	600, 2ws, 10	8	100%	iTBS	LDLPFC (F3)	No serious adverse events reported
Li et al. [87]	China	<3 months	37/28	40/22	MMSE < 26	Age: >50; sex: 72.7%	1) TMS (n = 33); 2) Sham (n = 32)	1,000, 4ws, 20	8	90%	LF-TMS 1 Hz	Contralateral DLPFC, (F3, F4)	NI
Fregni et al. [88]	US	>1 year	3/12	Ischemic	Mild-to-moderate motor deficit	Age: 38-75; sex: 73.3%	1) TMS (n = 10); 2) Sham (n = 5)	1,200, 5ds, 5	8	100%	LF-TMS 1 Hz	Unaffected hemisphere	1 mild headache, 1 anxiety. Sham: 1 tiredness, 1 mild headache
Lu et al. [89]	China	<1 year	18/22	18/22	No severe aphasia or cognitive disorder	Age average: 44.9 ± 11.1; sex: 62.5%	1) LF-TMS + Computer Cog training (n = 19); 2) Sham + Computer cog training (n = 21)	600, 4ws, 20	NI	100%	LF-TMS 1 Hz	RDLPFC	TMS: 1 transient headache, 1 dizziness; Sham: 1 headache

In terms of intervention, the total pulses in one session ranged from 450 to 2,000 pulses; of these, 600 pulses had the highest number of RCTs (six RCTs). Almost all the RCTs used eight-shaped coils. Seven RCTs used HF-TMS, four RCTs used LF-TMS, and four RCTs used iTBS in the intervention group. The intensity ranged from 70% to 100% of the motor threshold. Regarding the stimulation positions, 10 of 13 RCTs used the LDLPFC, one RCT used the RDLPFC, one RCT used the contralateral side, and one RCT used the unaffected side (all three RCTs used 1 Hz). Five studies mentioned adverse effects; however, no significant adverse effects were observed. None of the studies report any conflicts of interest.

Outcome Measures

With the outcome measures related to executive function, attention, and memory, some studies provided a subscore of the Montreal Cognitive Assessment (MoCA), Assessment of Neuropsychological Status (RBANS), LOTCA, and the Oxford Cognitive Screen (OCS), while some other studies provided a scale for assessing these outcomes (Table 2) including the following: 1) Executive function: N-back task (1RCT) and backward digit span (three RCTs Backward DS) for working memory, Tower of London test, Word/Color of color word test, Stroop color-word test, Stroop test, Victoria Stroop Test (VST), SubMoCA, and SubOCS; 2) Attention: Forward digit span (3RCT), auditory continuous performance test CPT (seconds) (1RCT), Visual CPT (seconds), Trail Making Test-A (1RCT), subRBANS (1RCT), subLOTCA (1RCT); and subMoCA; and 3) Memory: N-back task (1RCT) and backward digit span (three DS Forward) for working memory, Rivermead behavioral memory test (three) (3RCTs), digit symbol test (1RCT), verbal learning test (1RCT), visual learning test (1RCT), subRBANS (1RCT), subOCS (1RCT), and subMoCA.

**Table 2:** Outcome measures of TMS on executive function, attention, and memory LOTCA: Loewenstein Occupational Therapy Cognitive Assessment; MoCA: Montreal Cognitive Assessment; OCS: Oxford Cognitive Screen; OCS: Oxford Cognitive Screen; RBANS: Assessment of Neuropsychological Status; RBMT: Rivermead Behavioral Memory Test; sub: subdomain; SCWT: Stroop color and word test; TMS: transcranial magnetic stimulation; TMT-A: Trail Making Test-A Victoria Stroop Test includes three parts: A, B, C ^a^Working memory test ^1^Nonnormal distribution

Study	Outcome measures		
	Executive function	Attention	Memory
Najafabadi et al. [78]	N-back task^a^	-	N-back task^a^
Hu et al. [79]	-	-	RBMT
Kim et al. [80]	Word of color word test; color of color word test; Tower of London; Backward Digital Span Test^a^	Visual/auditory continuous performance test; Forward Digital Span Test	Verbal learning test; visual learning test; Backward Digital Span Test^a^
Zhang et al. [81]	-	LOTCA^1^ (sub)	-
Yu et al. [82]	SCWT	-	-
Yin et al. [83]	MoCA (sub); Victoria Stroop Test (A^1^, B^1^, C)	MoCA^1^ (sub)	MoCA^1^ (sub); RBMT
Liu et al. [84]	Backward Digital Span Test^a^	TMT-A; Forward Digital Span Test	Digit Symbol Test; Backward Digital Span Test^a^
Chu et al. [53]	-	LOTCA (sub)^1^	-
Tsai et al. [85]	-	RBANS (sub)	RBANS (sub)
Li et al. [86]	OCS^1^ (sub)	-	OCS^1^ (sub)
Li et al. [87]	MoCA^1^ (sub)	MoCA (sub)	MoCA (sub)
Fregni et al. [88]	Stroop test; Backward Digital Span Test^a^	Forward Digital Span Test	Backward Digital Span Test^a^
Lu et al. [89]	-	-	RBMT
Total article	8	8	10

Risk of Bias Assessment

**Figure 2:**
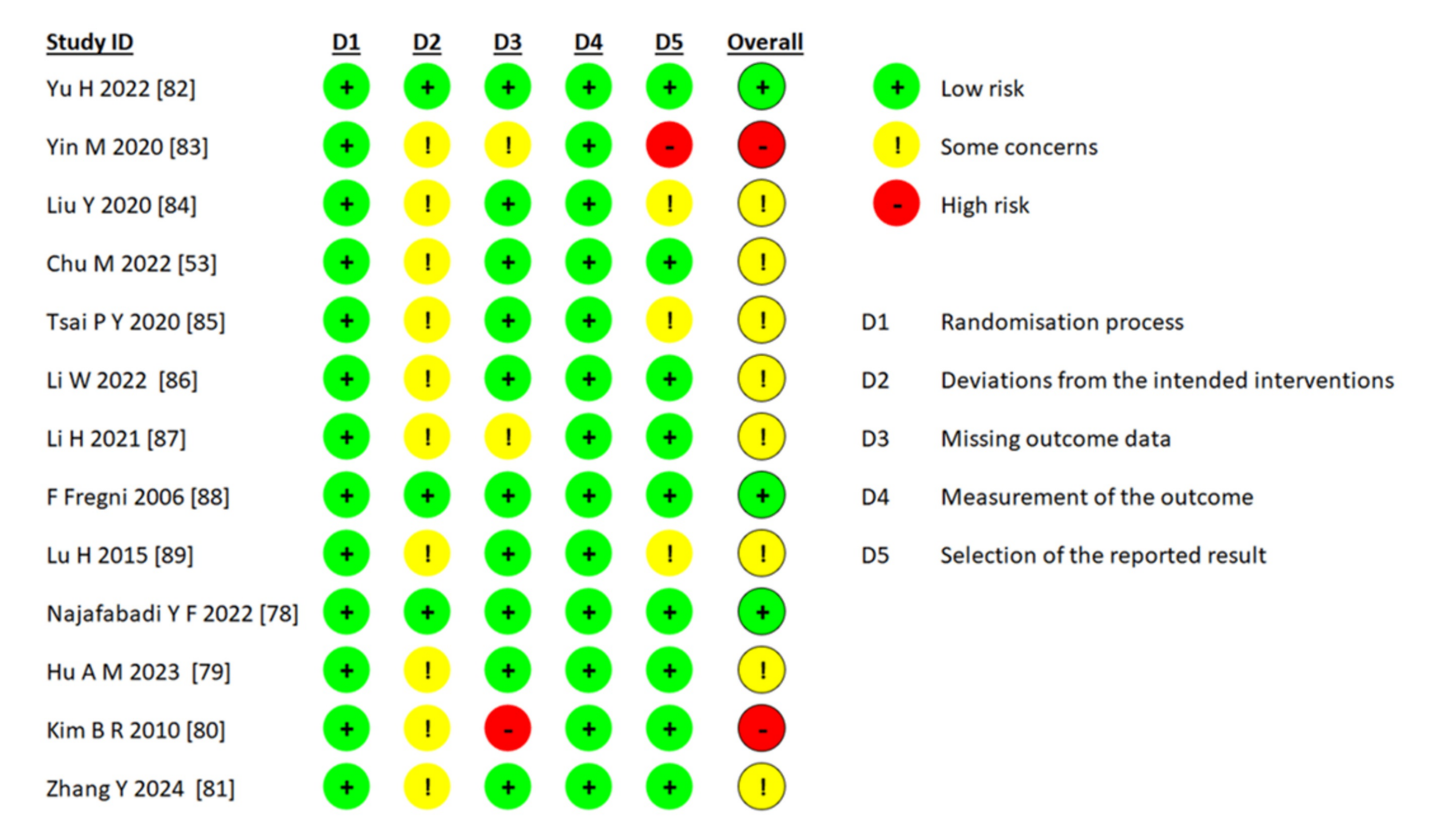
Risk of bias summary of determinations for each risk of bias domain (D) Image credits: This is an original image created by the author Ha T. Le

The risk of bias ratings for all 13 studies in accordance with the Cochrane Handbook guidelines are shown in Figure 2.

Two studies were rated as having a high risk of bias [80,83], eight studies were considered to have some concerns [53,79,81,84-87,89], and three studies were evaluated with a low risk of bias [78,82,88]. The reasons for downgrading (with the number of downgraded RCTs decreasing, respectively) were deviations from the intended interventions, selection of the reported result, missing outcome data, and randomization process. All 13 studies were judged to have a low risk of bias in the outcome domain measurements. Two high-risk studies were included due to high risk in the randomization process, missing outcome data, and deviations from the intended interventions.

Effects of TMS

In demonstrating the effect of TMS on executive function, attention, and memory, 13 RCTs used different outcome measures. Regarding executive function, six studies with meta-analyses showed significant effectiveness. A meta-analysis of nine studies showed that TMS significantly improved memory function. Among the attention outcomes, five studies considered moderate effectiveness but were not significant.

**Figure 3:**
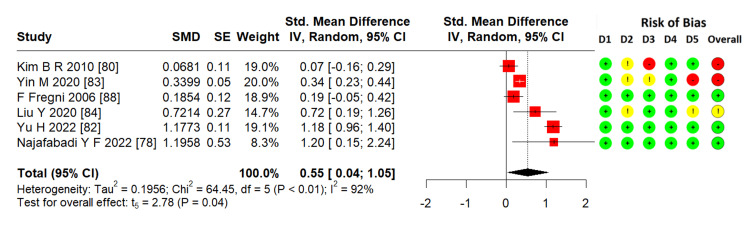
Forest plot of effectiveness of TMS on executive function SMD: standardized mean difference; SE: standard error; IV: inverse variance; CI: confidence interval; TMS: transcranial magnetic stimulation Image credits: This is an original image created by the author Ha T. Le

Effects of TMS on executive function: From the eight studies [78,80,82-84,88], six RCTs (161 patients) were pooled to calculate the ES using a meta-analysis. TMS significantly enhanced executive function in patients with stroke (SMD = 0.55; 95% CI = 0.04-1.05; p = 0.04; I² = 92%) (Figure 3).

**Figure 4:**
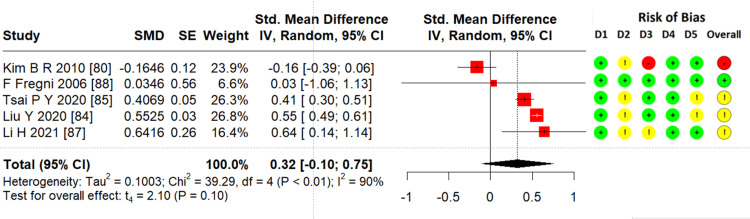
Forest plot of effectiveness of TMS on attention SMD: standardized mean difference; SE: standard error; IV: inverse variance; CI: confidence interval; TMS: transcranial magnetic stimulation Image credits: This is an original image created by the author Ha T. Le

Effects of TMS on attention: From the eight studies [53,80,81,83-85,87,88], five RCTs (n = 197) were pooled to estimate the ES. TMS did not significantly alleviate attention function in stroke patients (SMD = 0.32; 95% CI = -0.1 to 0.75; p = 0.1; I² = 90%) (Figure 4).

**Figure 5:**
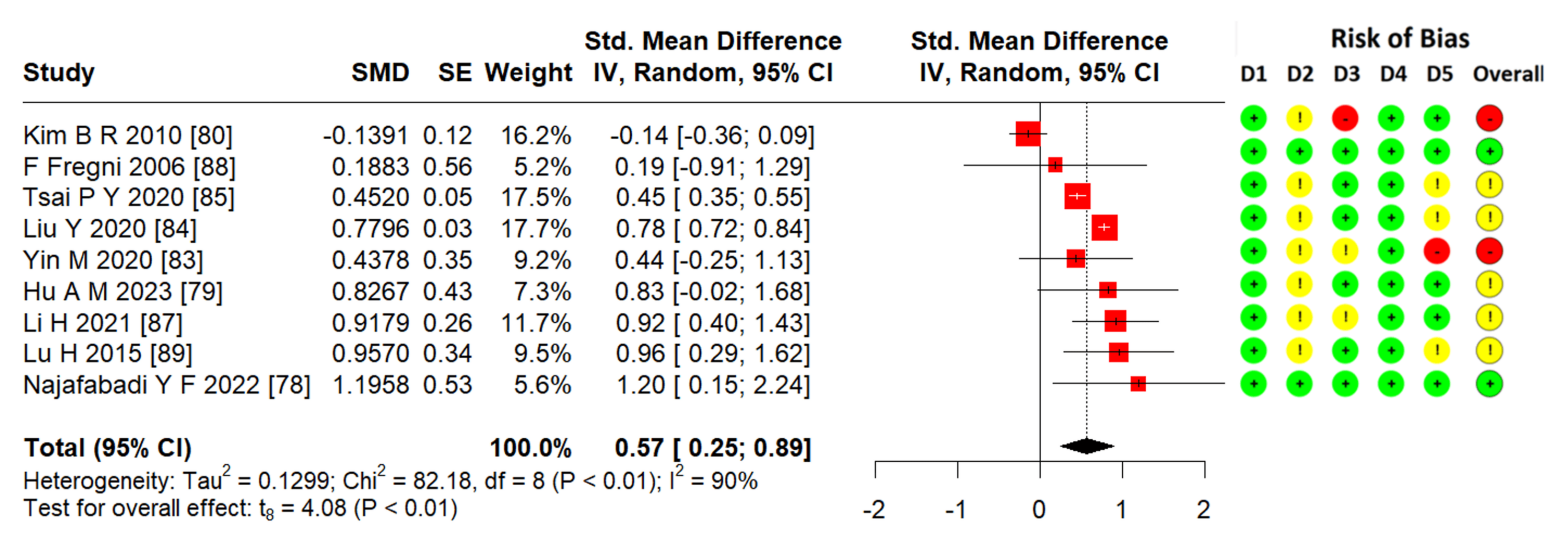
Forest plot of effectiveness of TMS on memory SMD: standardized mean difference; SE: standard error; IV: inverse variance; CI: confidence interval; TMS: transcranial magnetic stimulation Image credits: This is an original image created by the author Ha T. Le

Effects of TMS on memory: From 10 studies [78-80,83-89], nine RCTs (n = 323) were pooled in a meta-analysis to estimate the ES. TMS showed significant efficacy in improving memory function in stroke patients (SMD = 0.57; 95% CI = 0.25-0.89; p < 0.01; I² = 90%) (Figure 5).

Certainty of the Evidence

The quality of evidence for the problems is shown in Table 3, according to the GRADE assessment. The results of the effectiveness of TMS in the subdomains of cognition showed low-quality to very low-quality evidence for all outcomes. The memory outcomes showed low-quality evidence because of the risk of bias and imprecision owing to the small sample size. Executive function and attention were assessed using low-quality evidence because of inconsistencies and the risk of bias.

**Table 3:** Effect of TMS on executive function, attention, and memory BDS: backward digital span test; CCWT: color of color word test; CPT: continuous performance test; DST: digit symbol test; FDS: forward digital span test; GRADE: Grading of Recommendations Assessment, Development, and Evaluation; MoCA: Montreal Cognitive Assessment; N-BT: N-back task; RBANS: assessment of neuropsychological status; RBMT: Rivermead behavior memory test; RCTs: randomized controlled trials; SCWT: Stroop color and word test; SMD: standardized mean difference; ST: Stroop test; sub: subgroup; TMT-A: trail making test-A; verbal LT: verbal learning test; visual LT: visual learning test; VST: victoria Stroop test; WCWT: word of color word test ^a^Downgraded one level due to risk of bias ^b^Downgraded one level due to inconsistency ^c^Downgraded one level due to imprecision

Outcomes	Results	No. of participants (studies)	Certainty of evidence (GRADE)	Comments
Executive function assessed with: N-BT, BDS, CCWT, WCWT, Tower of London test, SCWT, ST, VST Follow-up: two weeks (FDS, ST)	SMD 0.55 higher (0.04 higher to 1.05 higher)	161 (6 RCTs)	⨁◯◯◯ Very low^a,b,c^	TMS might improve executive function
Attention assessed with: FDS, auditory CPT, visual CPT, TMT-A, subRBANS, subMoCA follow-up: 2 weeks (forward digit span)	SMD 0.32 higher (0.1 lower to 0.75 higher)	197 (5 RCT)	⨁◯◯◯ Very low^a,b,c^	Uncertain about the effect of TMS on attention. More evidence is needed
Memory assessed with: N-BT, BDS, RBMT, DST, Verbal LT, Visual LT, subRBANS, subMoCA Follow-up: 2 months (RBMT)	SMD 0.57 higher (0.25 higher to 0.89 higher)	323 (9 RCT)	⨁⨁◯◯ Low^b,c^	TMS might improve memory

Subgroup Analysis

Subgroup analyses were conducted based on the type of TMS and the time of onset. The results showed that HF-TMS significantly affected executive function and memory, and for time of onset, TMS improved in patients with a time of onset of under one year (see Appendix 2). All outcomes of the analysis were judged as very low-to-moderate evidence (see Appendix 3).

Discussion

This systematic study aimed to collect evidence of the effects of TMS on executive function, attention, and memory in patients with stroke. The results showed the effectiveness of TMS on memory with low quality and executive function with very low quality. However, there was no significant impact on attention with very low quality using the GRADE system. HF-TMS, iTBS, and early intervention showed superior results. The evidence for these results is discussed in greater detail below.

Executive Function

The impairment of executive functions includes many aspects, such as attention flexibility, working memory with the ability to update information, initiation, processes of planning, organization, inhibition, self-monitoring, problem solving, and error correction, or could be divided into three subdomains: shifting, real-time monitoring and updating, and inhibition. They are essential for responding to novel and new situations and goal-oriented behaviors [9,90-92]. Furthermore, they are frequently affected in stroke patients [9], resulting in a disability in regaining independence in daily life and predicting functional recovery [91,93]. This review conducted a meta-analysis of six RCTs that showed significant effectiveness (Figure 3), with very low evidence following the GRADE approach. To the best of our knowledge, there has been no systematic review or meta-analysis on the effect of TMS on executive function in patients with stroke. There were some systematic reviews that analyzed subgroups about the effect of TMS on the executive function, which also agree with this effectiveness of the analyzed working memory with two RCTs (one RCT with three groups) by Hara et al. [94] and with four RCTs (two RCTs used LOTCA, one RCT used VST, and one RCT used word of color word test, color of color word test, and Tower of London) [45]. This systematic review included all those RCTs but did not use LOTCA or delayed memory as outcomes measure for executive function because these tests do not really represent executive function. Han et al. [95] reported the same results in a subgroup analysis of two Chinese-language RCTs. In addition, some studies [62,70,71,75] were excluded because they were nonrandomized controls, published only abstracts, or because one group study mentioned the effect of TMS on executive function in stroke. These studies agree that TMS enhances executive function.

Attention

Attention deficits may be related to various aspects of the attention process [40] at various levels and incidences, including selective (35%), divided (41%), and sustained (31%) attention deficits [96]. These impairments affect everyday functioning, are serious obstacles to rehabilitation, and are related to difficulties in balance, falls, and daily living activities [96,97]. In this study, an attention meta-analysis was performed with five RCTs that presented moderate effectiveness but were insignificant (Figure 4). Thus far, there have been no systematic reviews on the effects of TMS on attention in patients with stroke. However, some systematic reviews have analyzed subgroups regarding the effect of TMS on attention, such as those by Li et al. [32], Yang et al. [34], Hara et al. [94], and Han et al. [95], which showed a significant effect of TMS on attention. Another subgroup analysis by Gao et al. with one two-arm RCT (three groups) showed no effect on attention [45]. However, this RCT had a small sample size of 18 patients in three groups with a high risk of bias. This review included all the above RCTs, with the exception of one Chinese-language RCT. The results showed effectiveness but not significance. Because of the risk of bias and small sample size, the results were assessed with very low evidence following the GRADE approach. Therefore, careful consideration is required when reading the results. Among the excluded studies, one nonrandomized study mentioned attention results, with 133 patients showing that HF-TMS enhanced attention when comparing the intervention and control groups [63].

Memory

There are three types of memories: long-term, short-term, and working [98]. People with memory problems after a stroke often experience difficulties in everyday life [99]. In this review, a memory meta-analysis was conducted using nine RCTs that showed significant effectiveness (Figure 5) with low evidence following the GRADE approach.

Some systematic reviews of cognitive impairment [32,34,94,95] that analyzed the effect of TMS on memory in stroke patients showed its effectiveness, all of which were analyzed in two to three RCTs. One subgroup of a systematic review [45] showed that TMS had no effect on memory with a meta-analysis of three RCTs; one included study with high risk and small sample size negatively affected the results, which should be carefully considered; all these RCTs were included in the studies of this review except two Chinese-language RCTs. Notably, two systematic reviews of Chinese-language RCTs on the effect of TMS on memory in patients with stroke [100,101] also showed a significant effect.

All three main meta-analyses showed heterogeneity (Figures 3-5) with I² > 90%. The possible reasons may be as follows. First, outcomes with five to nine RCTs were assessed by different scales and subscales (Table 2). As discussed, executive function, attention, and memory are contributed by some other components, and some scales and subscales might not comprehensively represent these subdomain's functions. Therefore, these outcomes were pooled for the meta-analysis. Second, not all studies have the same design (dose, type of TMS, time of onset, lesion, as shown in Table 1). The differing inclusion criteria contribute to the difference in results. In particular, executive function, attention, and memory assessment results were affected by many factors, including aphasia, time of onset, praxis, neglect, and hemispheric lesion [9,40,102,103]. This may have resulted in differences between the included studies. The above reasons also contributed to downgrading the quality of evidence in each outcome to very low, low, and moderate quality using the GRADE system (Table 3 and Appendix 3). There were three RCTs with a nonstandard distribution, and the data expressed as medians, which did not represent the research outcome, were excluded from the meta-analysis. Therefore, to draw strong conclusions, we need more evidence with highly rigorous quality and specific scales that better represent executive function, attention, and memory, especially with their components.

Almost all RCTs in the meta-analysis compared TMS with sham or control groups. Regarding the intervention, as mentioned above, the stimulation position and intensity were quite consistent between studies, and adverse effects were reported in five RCTs that were not dominant and different from the control group. Follow-up results showed efficacy on memory after two months and executive function after two weeks but not on attention.

Some subgroups were based on the type of TMS and time of onset to understand its effectiveness in more detail. Further analysis showed many results; notably, early intervention in the first year after onset, HF-TMS, and iTBS showed significant effectiveness on executive function and memory, which is consistent with the current evidence [34,39,104,105].

Limitations, Strengths, and Prospects

This review has some limitations: 1) it was limited to English studies and was performed in seven databases; therefore, it is not possible to identify all available evidence, and 2) because many factors have been discussed, the meta-analysis had a bias, reducing the evidence quality.

However, the study collected the best evidence regarding the effectiveness of TMS on executive function, attention, and memory, including discussions with related SRs. Multiple subgroups were conducted to contribute more comprehensively to the current evidence.

The results of this systematic review have important implications for TMS in executive function, attention, and memory after stroke. First, regarding research, 1) future research should be more rigorously effective in obtaining high-quality evidence, especially related to subdomains of cognition and components of these subdomains; 2) TMS should be investigated in the follow-up period, more specifically in the time of onset, and comprehensive scales for the assessment of executive function, attention, and memory deficits in stroke; and 3) almost all studies were stimulated in the LDLPFC; meanwhile, executive function, attention, and memory areas might be different. Therefore, more details regarding the intervention dose should be considered for each subdomain. Second, regarding clinical applications, due to its efficacy, safety, and good tolerance, TMS showed positive effects on executive and memory deficits. This intervention can be performed early in the recovery process after stroke, and HF-TMS showed better results.

## Conclusions

These results suggest that TMS affects executive function and memory. However, the attention outcomes did not show a clear benefit of TMS itself in general. One year after stroke, HF-TMS and iTBS were effective in improving executive function and memory. According to the GRADE system, the evidence level varies from very low to moderate. Owing to the heterogeneity, imprecision, potential risks of bias, and lack of data from the included trials, the results should be interpreted with caution. Furthermore, more rigorous evidence on the effectiveness of TMS in executive function, attention, and their components is needed.

## Figures and Tables

**Figure 6:**
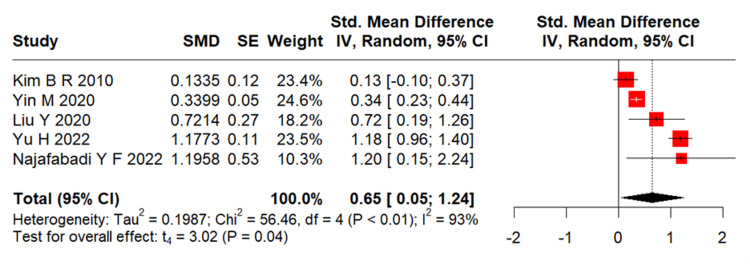
Effectiveness of high-frequency TMS on executive function SMD: standardized mean difference; SE: standard error; IV: inverse variance; CI: confidence interval; TMS: transcranial magnetic stimulation Image credits: This is an original image created by the author Ha T. Le

**Figure 7:**
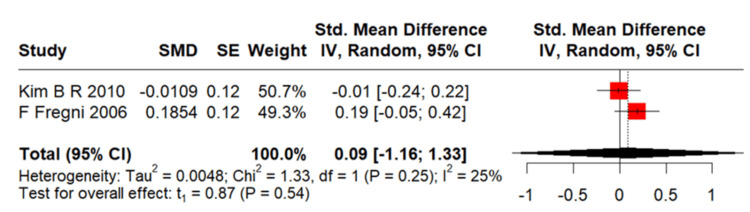
Effectiveness of low-frequency TMS on Executive function SMD: standardized mean difference; SE: standard error; IV: inverse variance; CI: confidence interval; TMS: transcranial magnetic stimulation Image credits: This is an original image created by the author Ha T. Le

**Figure 8:**
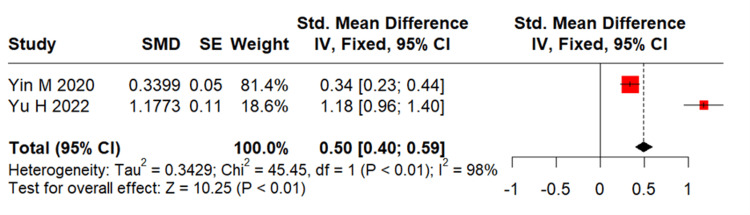
Effectiveness of TMS on Executive function (less than six months) SMD: standardized mean difference; SE: standard error; IV: inverse variance; CI: confidence interval; TMS: transcranial magnetic stimulation Image credits: This is an original image created by the author Ha T. Le

**Figure 9:**
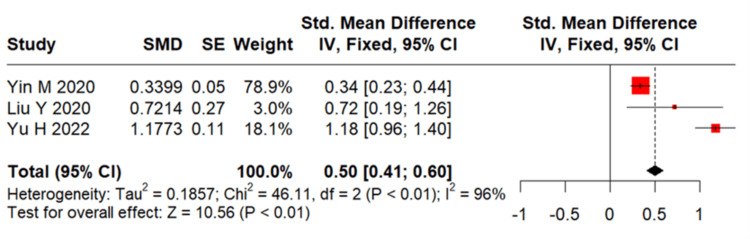
Effectiveness of TMS on Executive function (less than one year) SMD: standardized mean difference; SE: standard error; IV: inverse variance; CI: confidence interval; TMS: transcranial magnetic stimulation Image credits: This is an original image created by the author Ha T. Le

**Figure 10:**
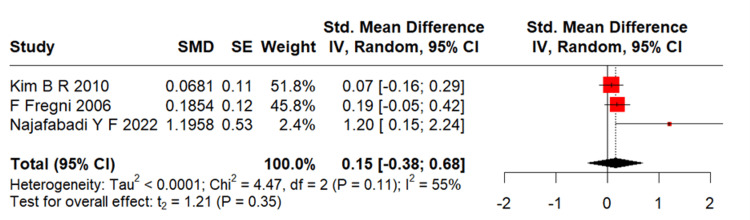
Effectiveness of TMS on Executive function (greater than one year) SMD: standardized mean difference; SE: standard error; IV: inverse variance; CI: confidence interval; TMS: transcranial magnetic stimulation Image credits: This is an original image created by the author Ha T. Le

**Figure 11:**
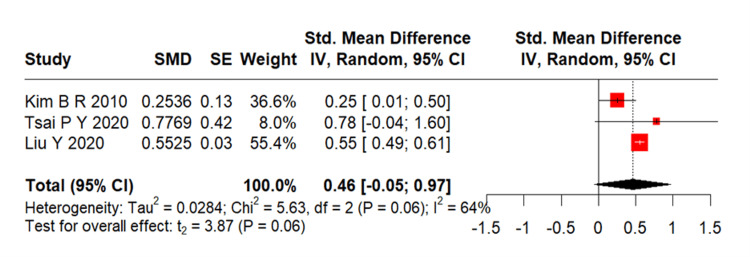
Effectiveness of high-frequency TMS on Attention function SMD: standardized mean difference; SE: standard error; IV: inverse variance; CI: confidence interval; TMS: transcranial magnetic stimulation Image credits: This is an original image created by the author Ha T. Le

**Figure 12:**
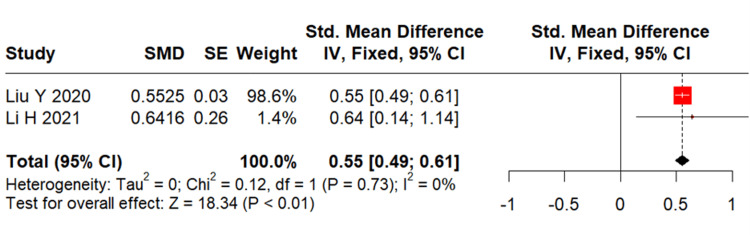
Effectiveness of TMS on Attention function (less than one year) SMD: standardized mean difference; SE: standard error; IV: inverse variance; CI: confidence interval; TMS: transcranial magnetic stimulation Image credits: This is an original image created by the author Ha T. Le

**Figure 13:**
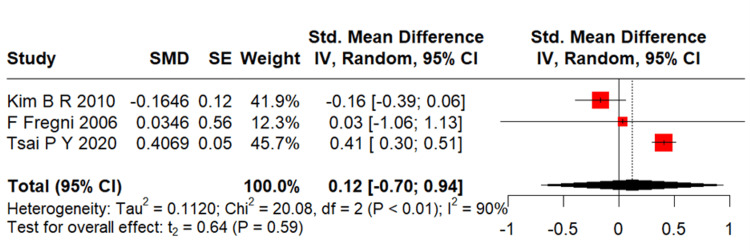
Effectiveness of TMS on Attention function (greater than one year) SMD: standardized mean difference; SE: standard error; IV: inverse variance; CI: confidence interval; TMS: transcranial magnetic stimulation Image credits: This is an original image created by the author Ha T. Le

**Figure 14:**
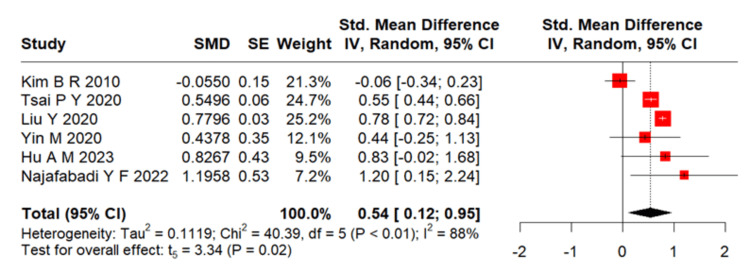
Effectiveness of high-frequency TMS on Memory function SMD: standardized mean difference; SE: standard error; IV: inverse variance; CI: confidence interval; TMS: transcranial magnetic stimulation Image credits: This is an original image created by the author Ha T. Le

**Figure 15:**
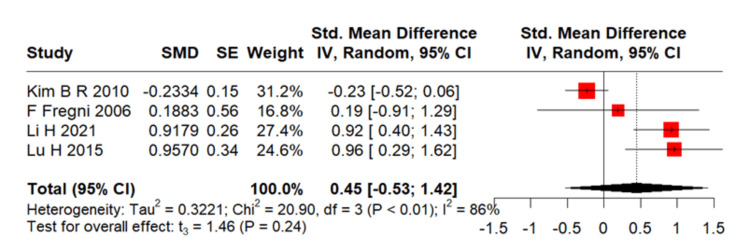
Effectiveness of low-frequency TMS on Memory function SMD: standardized mean difference; SE: standard error; IV: inverse variance; CI: confidence interval; TMS: transcranial magnetic stimulation Image credits: This is an original image created by the author Ha T. Le

**Figure 16:**
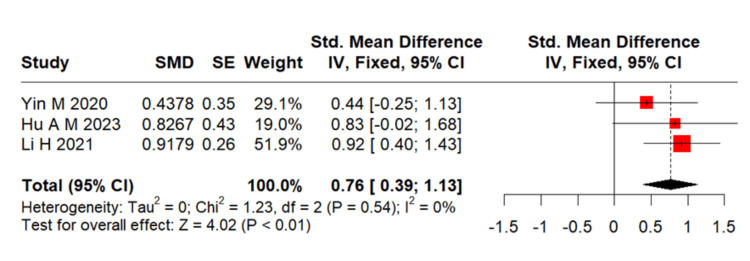
Effectiveness of TMS on Memory function (less than six months) SMD: standardized mean difference; SE: standard error; IV: inverse variance; CI: confidence interval; TMS: transcranial magnetic stimulation Image credits: This is an original image created by the author Ha T. Le

**Figure 17:**
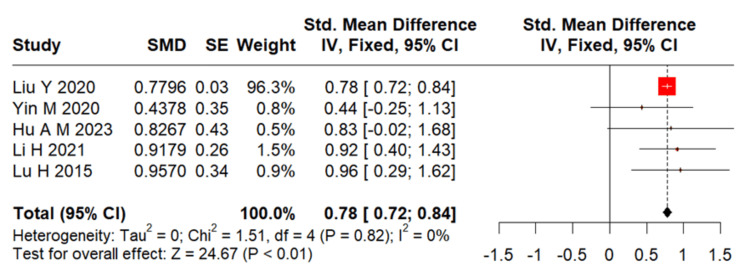
Effectiveness of TMS on Memory function (less than one year) SMD: standardized mean difference; SE: standard error; IV: inverse variance; CI: confidence interval; TMS: transcranial magnetic stimulation Image credits: This is an original image created by the author Ha T. Le

**Figure 18:**
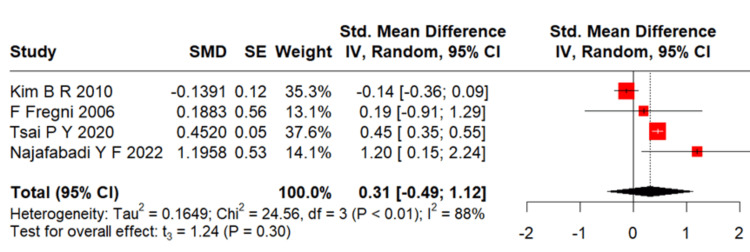
Effectiveness of TMS on Memory function (greater than one year) SMD: standardized mean difference; SE: standard error; IV: inverse variance; CI: confidence interval; TMS: transcranial magnetic stimulation Image credits: This is an original image created by the author Ha T. Le

## Appendices

Appendix 1

The following are the search strategies in seven databases (until July 31, 2024).

**Table 4:** Search strategy CINAHL: Cumulated Index in Nursing and Allied Health Literature; CVA: cerebrovascular accidents; CVA: cerebrovascular accidents; ICH: intracerebral hemorrhage; MeSH: Medical Subject Headings; rTMS: repettitive transcranial magnetic stimulation; SAH: subarachnoid hemorrhage; TMS: transcranial magnetic stimulation; MH: major heading

Search strategy
CENTRAL search strategy Cochrane Central Register of Controlled Trials (CENTRAL) search strategy #1 MeSH descriptor: [Cerebrovascular Disorders] explode all trees #2 [mh Basal Ganglia Cerebrovascular Disease] #3 [mh Brain Ischemia] #4 [mh Intracranial Embolism and Thrombosis] #5 [mh Intracranial Hemorrhages] #6 [mh Stroke] #7 [mh Hemiplegia] #8 (stroke* or “post stroke” or poststroke or post-stroke or apoplex* or “cerebral vascular disease” or cerebrovasc* or CVA or SAH or hemipar* or hemipleg* or paresis or paretic):ti,ab,kw #9 #1 or #2 or #3 or #4 or #5 or #6 or #7 or #8 #10 [mh Cognition Disorders] #11 [mh Cognitive Dysfunction] #12 [mh Cognition] #13 [mh Attention] #14 [mh Memory] #15 [mh Memory Disorders] #16 [mh Spatial Memory] #17 [mh Executive function] #18 (cognitive or cognition or attention* or inattention or concentrat* or memor* or recall or executive or dysexecutive):ti,ab,kw #19 (initiation or initiating or self-awareness or awareness or self-monitoring or self-inhibiting or self-evaluation or “goal management” or “goal selection” or “goal setting” or “goal-directed behavior” or “goal-directed behaviour” or “goal-directed activity” or “goal-directed activities” or “goal-directed movement” or “strategy formation” or planning or organisation or organization or organizing or reasoning or “time management” or “problem solving” or “decision making”) :ti,ab,kw #20 #10 or #11 or #12 or #13 or #14 or #15 or #16 or #17 or #18 or #19 #21 [mh Transcranial Magnetic Stimulation] #22 (TMS or rTMS or “Transcranial Magnetic Stimulation” or “theta-burst stimulation”):ti,ab,kw #23 #21 or #22 #24 #9 and #20 and #23 FILTERS: TRIALS, ENGLISH
PUBMED search strategy #1 “Stroke”[Mesh] OR “Stroke, Lacunar”[Mesh] OR “Hemorrhagic Stroke”[Mesh] OR “Embolic Stroke”[Mesh] OR “Thrombotic Stroke”[Mesh] OR “Ischemic Stroke”[Mesh] OR “Stroke Rehabilitation”[Mesh] OR “Infarction, Posterior Cerebral Artery”[Mesh] OR “Brain Stem Infarctions”[Mesh] OR “Infarction, Middle Cerebral Artery”[Mesh] OR “Infarction, Anterior Cerebral Artery”[Mesh] #2 (Title/Abstract): (stroke* or “post stroke” or poststroke or post-stroke or apoplex* or “cerebral vasc*” or cerebrovasc* or cva or SAH or hemipar* or hemipleg* or paresis or paretic) #3 #1 or #2 #4 (“Cognition”[Mesh] OR “Cognition Disorders”[Mesh] OR “Cognitive Dysfunction”[Mesh] OR “Attention”[Mesh] OR “Memory”[Mesh] OR “Spatial Memory”[Mesh] OR “Memory Disorders”[Mesh] OR “Executive Function”[Mesh]) #5 (cognitive or cognition or attention* or inattention or concentrat* or memor* or recall or executive or dysexecutive) (Title/Abstract) #6 (initiation or initiating or self-awareness or awareness or self-monitoring or self-inhibiting or self-evaluation or “goal management” or “goal selection” or “goal setting” or “goal-directed behavior” or “goal-directed behaviour” or “goal-directed activity” or “goal-directed activities” or “goal-directed movement” or “strategy formation” or planning or organisation or organization or organizing or reasoning or “time management” or “problem solving” or “decision making”) (Title/Abstract) #7 #4 OR #5 OR #6 #8 (TMS or rTMS or “Transcranial Magnetic Stimulation” or “theta-burst stimulation”) (Title/Abstract) #9 “Transcranial Magnetic Stimulation”[Mesh] #10 #8 or #9 #11 #3 AND #7 AND #10 FILTER: CLINICAL TRIAL AND RANDOMIZED CONTROLLED TRIAL
SCOPUS search strategy TITLE-ABS-KEY ( ( stroke OR {post stroke} OR poststroke OR post-stroke OR apoplex! OR “cerebral vasc!” OR cerebrovasc! OR cva OR sah OR hemipar! OR hemipleg! OR paresis OR paretic OR {Ischemic stroke} OR {cerebral infarction} OR {cerebral thrombosis} OR {lacunar stroke} OR {Cerebral hemorrhage} OR {intracerebral hemorrhage} OR ich OR {subarachnoid hemorrhage} OR {brain hemorrhage} ) AND ( cognitive OR cognition OR attention! OR inattention OR concentrate! OR memor! OR recall OR executive OR dysexecutive OR initiation OR initiating OR self-awareness OR awareness OR self-monitoring OR self-inhibiting OR self-evaluation OR {goal management} OR {goal selection} OR {goal setting} OR {goal-directed behaviour} OR {goal-directed behavior} OR {goal-directed activity} OR {goal-directed activities} OR {goal-directed movement} OR {strategy formation} OR planning OR organisation OR organization OR organizing OR reasoning OR {time management} OR {problem solving} OR {decision making} ) AND ( tms OR rtms OR {transcranial magnetic stimulation} OR {theta-burst stimulation} ) ) AND ( LIMIT-TO ( DOCTYPE , “sh” ) OR LIMIT-TO ( DOCTYPE , “cp” ) OR LIMIT-TO ( DOCTYPE , “ar” ) ) AND ( LIMIT-TO ( LANGUAGE , “English” ) )
CINAHL search strategy CINAHL search strategy EBSCO Title (TI) (((MH “Cerebrovascular Disorders+”) OR (MH “Basal Ganglia Cerebrovascular Disease+”) OR (MH “Cerebral Ischemia+”) OR (MH “Carotid Artery Diseases+”) OR (MH “Intracranial Arterial Diseases+”) OR (MH “Arteriovenous Malformations+”) OR (MH “Intracranial Embolism and Thrombosis+”) OR (MH “Intracranial Hemorrhage+”) OR (MH “Stroke+”) OR (MH “Vertebral Artery Dissections+”) OR (MH “Hemiplegia+”)) OR stroke* or “post stroke” or poststroke or post-stroke or apoplex* or “cerebral vasc*” or cerebrovasc* or cva or SAH or hemipar* or hemipleg* or paresis or paretic AND (cognitive or cognition or attention* or inattention or concentrat* or memor* or recall or executive or dysexecutive or initiation or initiating or self-awareness or awareness or self-monitoring or self-inhibiting or self-evaluation or “goal management” or “goal selection” or “goal setting” or “goal-directed behavior” or “goal-directed behaviour” or “goal-directed activity” or “goal-directed activities” or “goal-directed movement” or “strategy formation” or planning or organisation or organization or organizing or reasoning or “time management” or “problem solving” or “decision making”) AND ((TMS or rTMS or “Transcranial Magnetic Stimulation” or “theta-burst stimulation”) Advanced search: Boolean/phrase English Language Language: English
NeuroBITE search strategy NeuroBITE (previously PsycBITE) Language: English Keyword: transcranial magnetic stimulation or theta-burst stimulation Target area: Cognition/Mental (All) Neurological Group: Stroke / CVA (Cerebrovascular Accidents) Method: Randomized trial controls
OTseeker search strategy (Title/Abstract) stroke or poststroke or CVA or cerebral vascular accident OR Ischemic stroke or cerebral infarction or cerebral thrombosis or cerebral embolism or lacunar stroke OR Cerebral hemorrhage or intracerebral hemorrhage or ICH or subarachnoid hemorrhage or SAH or brain hemorrhage AND (cognitive or cognition or attention* or inattention or concentrat* or memor* or recall or executive or dysexecutive AND (TMS or rTMS or Transcranial Magnetic Stimulation or theta-burst stimulation)
Pedro search strategy Abstract & Title (2 searches) “Transcranial magnetic stimulation” *stroke “Theta burst stimulation” *stroke Method: clinical trial

Appendix 2: Meta-analysis of subgroups: effectiveness of TMS on executive function, attention, and memory in stroke patients

Executive Function

The following describes the effectiveness of types of TMS on executive function.

Effectiveness of iTBS on Executive function: no RCT

Effectiveness of TMS on Executive function based on time of onset

Attention

Effectiveness of types of TMS on Attention function

Effectiveness of iTBS on Attention function: 1 RCT [85]

Effectiveness of TMS on Attention function based on time of onset

Effectiveness of TMS on Attention function (<3 months): 1 RCT [87]

Memory

Effectiveness of types of TMS on Memory function

Effectiveness of iTBS on Memory function: 1 RCT [85]

Effectiveness of TMS on Memory function based on time of onset

Effectiveness of TMS on Memory function (<3 months): 1 RCT [87]

Appendix 3

The summary of the finding table for subgroup analysis is as follows.

**Table 5:** Effect of TMS on executive function, attention, and memory with subgroup analysis CI: confidence interval; ES: effect size; GRADE: Grading of Recommendations, Assessment, Development, and Evaluations; HF-TMS: high-frequency transcranial magnetic stimulation; iTBS: intermittent theta-burst stimulation; LF-TMS: low-frequency transcranial magnetic stimulation; RCT: randomized controlled trial; SMD: standardized mean difference ^*^p < 0.05 ^a^Downgraded one level due to risk of bias ^b^Downgraded one level due to inconsistency ^c^Downgraded one level due to imprecision

Outcomes	Subgroup analysis	SMD/ES	95% CI	I^2^	No of participants (studies)	Certainty of evidence (GRADE)	Comments
Executive function	HF-TMS^*^	SMD = 0.65	0.05 to 1.24	93%	5 RCTs (n = 140)	⨁◯◯◯ Very low^a,b,c^	HF-TMS might improve executive function
	LF-TMS	SMD = 0.09	-1.16 to 1.33	25%	2 RCTs (n = 27)	⨁◯◯◯ Very low^a,b,c^	The evidence is uncertain about the effect of LF-TMS on executive function
	iTBS^*^	ES = 1.84	1.21 to 2.46	-	1 RCT (n = 58)	⨁⨁⨁◯ Moderate^a^	iTBS might improve executive function
	<3 months^*^	ES = 1.18	0.96 to 1.4	-	1 RCT (n = 18)	⨁⨁⨁◯ Moderate^c^	TMS might improve executive function in the first three months after stroke
	<6 months^*^	SMD = 0.50	0.4 to 0.59	98%	2 RCTs (n = 52)	⨁◯◯◯ Very low^a,b,c^	TMS might improve executive function in the first six months after stroke
	<1 year	SMD = 0.50	0.41 to 0.6	96%	3 RCTs (n = 110)	⨁◯◯◯ Very low^a,b,c^	TMS might improve executive function in the first year after stroke
	>1 year	SMD = 0.15	-0.38 to 0.68	55%	3 RCTs (n = 51)	⨁◯◯◯ Very low^a,b,c^	The evidence is uncertain about the effect of TMS on executive function more than one year after stroke
Attention	HF-TMS	SMD = 0.46	-0.05 to 0.97	64%	3 RCTs (n = 96)	⨁◯◯◯ Very low^a,b,c^	The evidence is uncertain about the effect of HF-TMS on attention
	LF-TMS	SMD = 0.19	-0.84 to 1.23	70%	3 RCTs (n = 92)	⨁◯◯◯ Very low^a,b,c^	The evidence is uncertain about the effect of LF-TMS on attention
	iTBS	ES = 0.2	-0.52 to 0.93	-	1 RCT (n = 30)	⨁◯◯◯ Very low^a,b,c^	The evidence is uncertain about the effect of iTBS on attention
	<3 months^*^	ES = 0.64	0.1 to 1.1	-	1 RCT (n = 65)	⨁⨁◯◯ Low^b,c^	TMS might improve attention in the first three months after stroke
	<6 months	-	-	-	-	-	No RCT
	<1 year^*^	SMD = 0.55	0.49 to 0.61	0%	2 RCTs (n = 123)	⨁⨁◯◯ Low^a,c^	TMS might improve attention in the first year after stroke
	>1 year	SMD = 0.12	-0.7 to 0.94	90%	3 RCTs (n = 74)	⨁◯◯◯ Very low^a,b,c^	The evidence is uncertain about the effect of TMS on attention more than one year after stroke
Memory	HF-TMS^*^	SMD = 0.54	0.12 to 0.95	88%	6 RCTs (n = 197)	⨁◯◯◯ Very low^a,b,c^	HF-TMS might improve memory
	LF-TMS	SMD = 0.45	-0.53 to 1.42	86%	4 RCTs (n = 132)	⨁◯◯◯ Very low^a,b,c^	The evidence is uncertain about the effect of LF-TMS on memory
	iTBS^*^	ES = 0.47	0.25 to 0.69	-	1 RCT (n = 41)	⨁⨁◯◯ Low^a,c^	iTBS might improve memory
	<3 months^*^	ES = 0.91	0.4 to 1.43	-	1 RCT (n = 65)	⨁⨁◯◯ Low^a,c^	TMS might improve memory in the first three months after stroke
	<6 months^*^	SMD = 0.76	0.39 to 1.13	0%	3 RCTs (n = 133)	⨁⨁◯◯ Low^b,c^	TMS might improve memory in the first six months after stroke
	<1 year^*^	SMD = 0.78	0.72 to 0.84	0%	5 RCTs (n = 231)	⨁⨁◯◯ Low^b,c^	TMS might improve memory in the first year after stroke
	>1 year	SMD = 0.31	-0.49 to 1.12	88%	4 RCTs (n = 92)	⨁◯◯◯ Very low^a,b,c^	The evidence is uncertain about the effect of TMS on memory more than one year after stroke
